# Randomized Controlled Trial Comparing the Effects of Far-Infrared Emitting Ceramic Fabric Shirts and Control Polyester Shirts on Transcutaneous PO_2_

**DOI:** 10.4172/2165-8064.1000349

**Published:** 2018-04-10

**Authors:** K Washington, J Wason, MS Thein, LA Lavery, MR Hamblin, IL Gordon

**Affiliations:** 1 Hologenix LLC, 1112 Montana Avenue, Santa Monica, California, USA; 2 Maelor Group 7 Village Woods Dr. Amherst, Massachusetts, USA; 3 Veterans Affairs Long Beach Healthcare System 5901 E. 7th Street, Long Beach California, USA; 4 University of Texas Southwestern 1801 Inwood Road Dallas, Texas, USA; 5 Wellman Centre for Photo-medicine, Massachusetts General Hospital, Harvard Medical School, Boston, Massachusetts 02114, USA; 6 Veterans Affairs Long Beach Healthcare System 5901 E. 7th Street, Long Beach California, USA

**Keywords:** FIR-emitting garments, Randomized controlled trial, Ceramic-embedded fabric, Ceramic-embedded clothing, Far-infrared radiation, Tissue oxygenation, Transcutaneous PO_2_, Donning sequence

## Abstract

Our aim was to confirm earlier studies showing tcPO_2_ to be higher under clothing made with polyethylene terephalate (PET) fabric containing ceramic particles (CEL) compared to standard PET fabric. In previous studies PET garments were donned first to avoid possible persistent effects from ceramic particles. This study randomized donning sequence to avoid bias.

**Methods:**

Subjects were randomized to don either PET shirts first (PETF n=73) or CEL first (CELF n=80), switching garments after 90 minutes. Skin temperature (ST), arterial oxygen saturation (O_2_sat), and tcPO_2_ were measured every 30 minutes.

**Results:**

Baseline ST and O_2_ sat were nearly identical in the two groups. Baseline tcPO_2_ was modestly higher in the CELF group than with PETF: 66.4 ± 18.9 vs. 63.9 ± 18.8 mmHg (n.s). Independent of donning sequence, tcPO_2_ measurements 90 minutes after wearing CEL were 6.7% higher than after 90 minutes wearing PET (p<0.0003). Sequence analysis found tcPO_2_ in PETF subjects to gradually rise before and after switching garments, but tcPO_2_ fell immediately after switching garments in CELF subjects. PETF baseline O_2_sat of 98.1 ± 1.3 increased insignificantly after 90 minutes, and then increased further to 98.6 ± 0.8 after wearing CEL ninety minutes (p=0.0001). CELF baseline O_2_sat of 97.9 ± 1.7 increased to 98.5 ± 1.1 90 minutes after donning CEL (p=0.0002) and fell to 98.3 ± 1.0 ninety minutes after switching to PET (p=0.0033).

**Conclusions:**

The ability of ceramic-embedded fabric to induce higher tcPO_2_ measurements is not due to sequence bias.

## Background

Far-infrared radiation (FIR) therapy has been used for the treatment of a variety of diseases and conditions [1-3], including pain [4-6], wound healing [7], recovery from exercise [8], heart failure [9], and disturbed sleep [10]. Often the FIR is delivered from an electrically-powered device such as an infrared heat lamp, an infrared sauna or a tourmaline/ jade heating pad. However, there is an alternative way to deliver FIR to the body, which is by the wearing of clothing constructed from ceramic particle-embedded fibers that emit FIR when powered by the wearer's own body heat [3]. Celliant^®^ (CEL, Hologenix LLC, Santa Monica, CA) yarn for clothing is constructed from standard polyester (polyethylene terephthalate --PET) extruded molten from a two-barrel machine with a proprietary mixture of ceramic particles added to produce a bicompatible fiber, with the center-load containing approximately 1 micron diameter particles [11]. CEL has been designed to capture heat from the body and re-emit radiant far-infrared energy to induce health benefits, and is thought to improve sleep and speed recovery from exercise. In one randomized clinical trial CEL socks were found to decrease chronic foot pain [12]. Tree previous unpublished studies examined the effects of CEL garments on transcutaneous partial pressure of oxygen (tcPO_2_) using Clark electrodes placed on the skin underneath either control PET garments or active CEL garments (http://www.pureenergysleep.ca/ clinical-studies/). Lavery in 2003 studied 20 subjects with diabetes and peripheral vascular disease wearing socks and gloves made from PET or CEL while resting quietly. With PET socks, foot tcPO_2_ levels fell on average 2.6% from baseline over 60 minutes, compared to an increase of 4.7% after 60 minutes wearing CEL socks. Hand tcPO_2_ increased 15.4% while wearing PET gloves for 60 minutes, compared to a significantly greater increase (30.8%) wearing CEL. A study in 13 normal subjects (McClue and Lavery, 2005, unpublished) compared tcPO_2_ values of the hands and feet after wearing either PET or CEL garments. One hour of wearing CEL gloves induced mean tcPO_2_ values in the hand to be 25.0% higher than those found with PET. Foot tcPO_2_ was 10.2% higher under CEL socks than under PET socks; both hand and foot differences were significant.

Gordon in 2009 measured tcPO_2_ in 24 healthy volunteers wearing either PET or CEL shirts. After one hour the tcPO_2_ was 7.1% higher under CEL shirts compared to PET shirts (p<0.05). No significant differences in mean blood pressure, heart rate, or temperature were found comparing measurements obtained while wearing CEL or PET. Due to a possible concern that the effects of CEL might persist after switching from active CEL garments to PET control garments, in these studies the PET garments were always worn first, before switching to CEL garments. This protocol could induce bias if, for example, tcPO_2_ steadily rises in resting subjects independent of fabric type. The current study compared measurements of skin temperature (ST), arterial oxygen saturation (O_2_sat) and tcPO_2_ obtained while subjects wore PET shirts or CEL shirts. In one group PET shirts were worn first and in the second CEL garments were worn first, with the order sequence being randomized. Our goal was to confirm earlier findings without risk of bias from influences of the sequence of wearing.

## Methods

**Figure 1:**
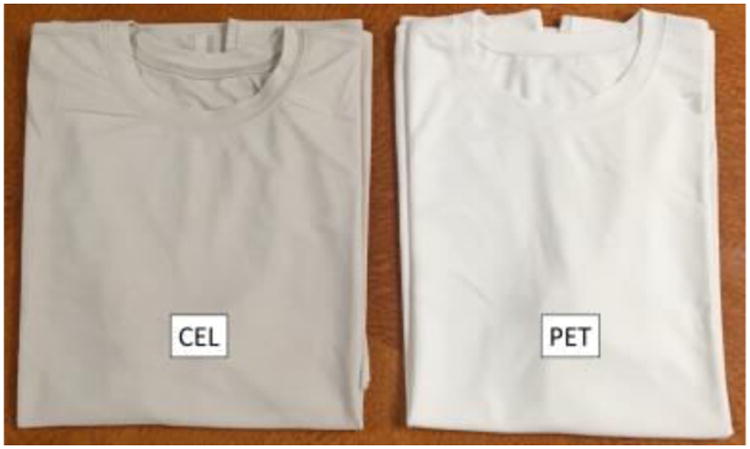
Appearance of CEL and PET shirts.

Between October 2013 and January 2014, healthy volunteers aged 18 to 60 were recruited for an IRB approved protocol via on-line advertisement for subjects that paid $25. The trial was registered at clinicaltrials.gov NCT02798640 (https://clinicaltrials.gov/ct2/show/ NCT02798640). The protocol was in accordance with the Declaration of Helsinki and informed consent was obtained. Exclusion criteria included cardiovascular disease, smoking, recreational drug use within 6 months, pregnancy, or consumption of alcohol within 48 hours or caffeine within 4 hours of enrolment. The garments employed were short sleeved shirts. PET and CEL garments were constructed at the same mill using either standard PET fiber or CEL fiber containing 1.25 % (mass ratio) proprietary ceramic particles. CEL and PET shirts were of identical fabrication and dernier differing only in color: PET was white and CEL was a light grey color (Figure 1).

Subjects were blinded to shirt composition and donning sequence. Assignment to either donning CEL shirts first (CELF) or control PET shirts first (PETF) was based 1:1 on a random allocation table. Studies were performed at the same constant temperature (24.0 ± 0.88°C), in a constant humidity (37.1 ± 5.0%) room with overhead fluorescent lighting. Prior to donning the first shirt, if necessary the anterior right shoulder skin was shaved, followed by gentle application of a fine abrasive and cleaning with tape and isopropyl alcohol. A probe with an electrolyte fluid ring, Clark electrode and a heating element set to heat the skin to 44°C was applied attached to a Periflux System 5000 monitor (Perimed, Kings Park, New York) with continuous monitoring software (Perisoft v2.55) as previously described [12]. Left forearm volar skin temperature (ST) was determined with an infrared thermometer. Fingertip pulse oximeter probes (CMS50DL, Crucial Medical Systems, and Atlanta, Georgia) monitored arterial oxygen saturation (O_2_sat). After preparation, subjects donned the first shirt, and sat quietly. Subjects were allowed to read or play video games but not sleep or converse.

Recordings of ST, O_2_sat, and tcPO_2_ began to be collected once tcPO_2_ measurements were deemed stable, which generally required 10 to 15 minutes. The tcPO_2_ signal representing mean ± s.d. was sampled at 32 Hz over 5 minutes. If s.d. was > 2 mmHg, the data was rejected as unstable. Cases in which the electrode membrane had to be replaced during measurements were also rejected. In 23 subjects data was excluded for one of these two problems, but all 23 returned on a later day to successfully repeat the entire protocol. No other data was excluded and all enrolled subjects completed the protocol.

Once the initial tcPO_2_ signal stabilized, baseline measurements were obtained followed by recordings 30, 60, and 90 minutes after baseline (A30, A60. A90). After the A90 measurement, subjects were encouraged to walk about, relieve themselves if necessary, and consume water and/or a small snack. The break interval was approximately 15 minutes. After the break, subjects donned the second shirt and resumed quiet sitting. A second set of measurements was obtained 30, 60, and 90 minutes after tcPO_2_ stabilized (B30, B60 and B90). Sample size was based on measurement of the variance in tcPO_2_ in 45 subjects with power analysis indicating that a minimum of 147 subjects would be required to achieve a 95% confidence level and power of 80%. Statistical analyses were performed only after all data had been acquired with subgroup analysis based only on gender and donning sequence.

## Results

Table 1 shows the demographics of the study population. The subjects were 54.3% male; 62.5% of the CELF sequence and 45.2% of the ConF sequence subjects were male (p=0.047, chi square test with Yates' correction). The age, height, weight, and BMI distribution were similar in the CELF and ConF subjects with no significant differences. Overall 52% of the subjects were Caucasian, 22% African-American, 16% Hispanic, 8% Asian, and 1% other – the ethnic distributions were comparable in the active and control sequences (53, 18, 20, 9, and 1% versus 52, 26, 20, 9, and 1%) (Table 1).

As shown in Table 2 is the baseline physiologic parameters. Mean baseline skin temperatures were nearly identical in the CELF and PETF groups 33.1 ± 0.9°C for CELF and 33.2 ± 1.1°C for PETF, as were baseline tcPO_2_ and O_2_sat. There were gender differences in baseline physiologic parameters: men had higher ST, lower O_2_sat, and lower tcPO_2_ then women. Mean baseline tcPO_2_ was 2.5 mmHg higher in the CELF group compared to the PETF group, (66.4 ± 18.9 versus 63.9 ± 18.8 mmHg) but not significantly (p=0.424 unpaired t test) (Table 2).

Table 3 shows ST and O_2_sat measurements. The mean baseline ST for all subjects was 33.1 ± 1.0°C, significantly higher (p<0.0001, paired t test) than mean 32.7± 1.1°C ST for all combined B90 measurements (the last measurements in each groups (data not shown). The difference between baseline and final B90 ST was significant in the CELF group (p<0.0001, paired t test) but not in the PETF group.

In contrast to the fall in ST observed from beginning to end of testing, O2sat increased from beginning to end. Combining all subjects, the mean baseline O_2_sat was 98.0 ± 1.3 compared to a mean value of 98.4 ± 0.9 for all B90 measurements (data not shown, p<0.0001, paired t test). Mean O_2_sat for all subjects after wearing CEL 90 minutes was significantly higher than O_2_sat for all subjects wearing PET 90 minutes (98.5 ± 0.8 vs. 98.2 ± 1.0, p<0.0001, paired t test). In PETF subjects, mean O_2_sat was 98.1 ± 1.3 at baseline and increased insignificantly (p=0.30 paired t test) to 98.2 ± 1.2 after ninety minutes, and then increased further to 98.6 ± 0.8 after wearing CEL shirts for ninety minutes (p=0.0001, paired t test). In the CELF group, however, mean baseline O_2_sat of 97.9±1.7 increased significantly to 98.5 ± 1.1 (p=0.0002, paired t test) after wearing CEL 90 minutes, then fell to 98.3 ± 1.0 after wearing PET 90 minutes (p=0.0033, paired t test) (Table 3).

**Figure 2:**
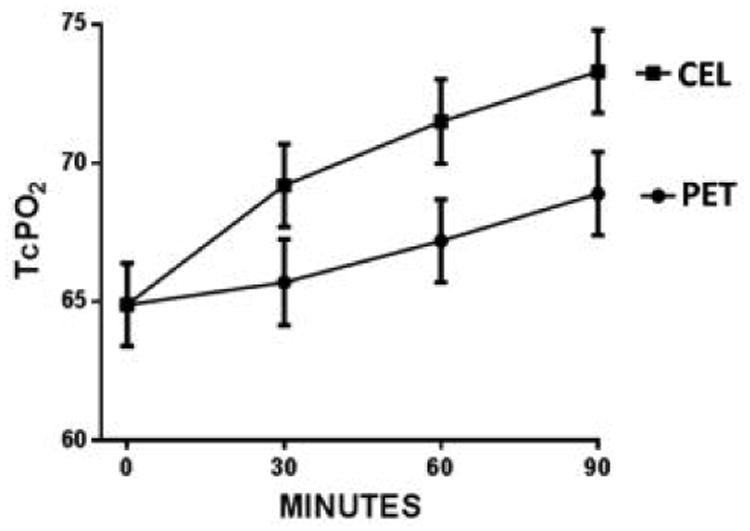
Combined tcPO_2_ measurements for PET and CEL garments independent of donning sequence.

Figure 2 shows tcPO_2_ for all the subjects combined, independent of donning sequence. Plotted are mean tcPO_2_ 30, 60, and 90 minutes after donning each garment-the baseline value is the mean for all subjects. At each interval tcPO_2_ is significantly higher for CEL compared to controls (p ≤ 0.0003, paired t tests). At 30 minutes, the difference between CEL and PET tcPO_2_ levels was 3.42 ± 11.29 mmHg (5.5%); at 60 minutes 4.30 ± 10.16 mmHg (6.4%); and at 90 minutes 4.44 ± 9.51 mmHg (6.7%). Each point represents the combined mean ± s.e.m tcPO_2_ for all subjects at the specified time interval after establishment of stable tcPO_2_ measurements after donning PET or CEL shirts (Figure 2).

**Figure 3:**
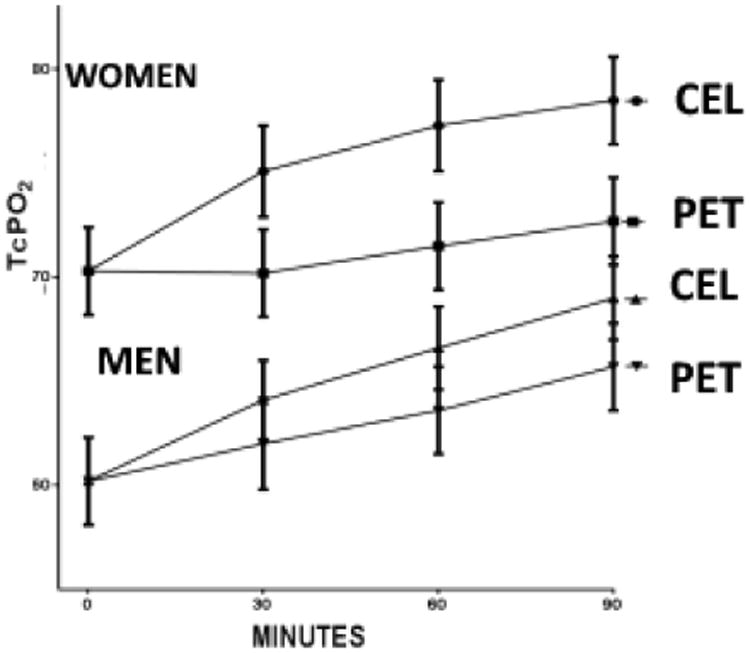
TcPO_2_ measurements based on gender.

Figure 3 shows the same analysis performed comparing female and male subjects' responses to PET and CEL-the same pattern was observed, but the differences in tcPO_2_ levels based on garment worn were more pronounced in women. Each point represents mean values ± s.e.m combining data from one gender at the specified time interval after establishment of stable tcPO_2_ measurements (Figure 3).

**Figure 4:**
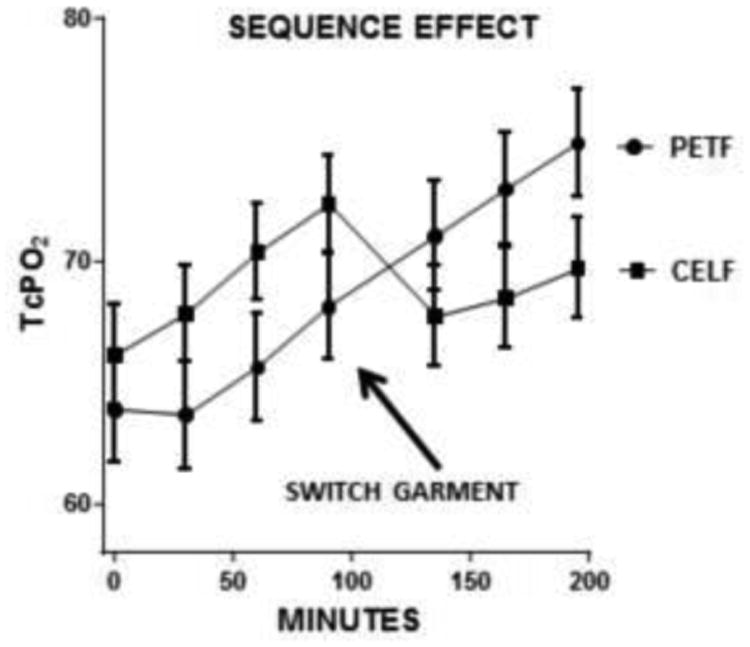
TcPO_2_ based on donning sequence.

Figure 4 shows the results of separating tcPO_2_ measurements based on donning sequence. The CELF and PETF baseline values are different. Mean tcPO_2_ is plotted in the temporal sequence with which it was measured. The PETF plot shows 30, 60, and 90 minute measurements obtained under PET shirts followed by 30, 60, and 90 minute measurements under CEL shirts. The plot for CELF subjects has the temporal sequence reversed: measurements obtained under CEL shirts are plotted before PET measurements. In the PETF group tcPO_2_ rose from baseline through A90 and continued to increase after switching garments. In contrast, tcPO_2_ levels in the CELF group increased through the A90 measurement but then fell after switching to PET. When the differences between tcPO_2_ measurements at each time interval are separately compared within sequence groups, i.e., 30 minute tcPO_2_ values for PET versus CEL in the CELF group, without inclusion of data from the PETF group and vice versa, two separate trends emerge. In the PETF group, differences between tcPO_2_ levels under PET vs. CEL are highly significant (p<0.0001) for each 30-minute interval by paired t testing. In contrast, in the CELF group, differences in tcPO_2_ between CEL and PET garments at 30 and 60 minutes are not significant, but approach significance at 90 minutes (p=0.051). Data from male and female subjects were combined and depicted in the sequence with which they were obtained (Figure 4).

When the same sequence analysis was applied to each gender, the same patterns were seen: tcPO_2_ levels fell in both CELF male and female subjects after switching from CEL to PET shirts, but continued to increase in both male and female PETF subjects after switching from PET to CEL (data not shown).

## Discussion

This study measured changes in tcPO_2_ at 30 minute intervals up to 90 minutes using Clark electrodes placed under either PET and CEL shirts which differed only by the presence or absence of ceramic particles in otherwise identical PET fibers. Subjects were randomized to wear either PET or CEL garments first. In general, tcPO_2_ levels tended to increase while wearing either garment from baseline to end of the complete protocol. When tcPO_2_ measurements were combined without reference to the sequence with which garments were worn, measurements under CEL garments were 5.5% higher at 30 minutes and 6.7% higher at 90 minutes (p<0.05). A different pattern of tcPO_2_ measurements emerges, however, when tcPO_2_ is plotted based on donning sequence (Figure 4). In subjects who wore CEL first, tcPO_2_ fell immediately after switching to control PET garments, but the opposite result was seen in subjects who donned PET first--tcPO_2_ continued to rise after switching to CEL. The sequence data support the conclusion that wearing CEL is associated with greater increases in tcPO_2_ than PET, independent of donning sequence. Interestingly, the differences in tcPO2 measured at each interval between garments (e.g., mean A60 and mean B60 values) within the PETF group were significant, but not in the CELF group. This finding, in conjunction with the sequence pattern suggests that CEL effects might persist long enough to influence measurements taken after switching to PET. The 9.7% difference in mean tcPO2 levels between PET and CEL in the PETF group at 90 minutes (68.2 mmHg versus 74.9 mmHg) is comparable to results of the previous studies which employed that sequence.

CELF subjects had a mean baseline tcPO_2_ that was 2.5 mmHg higher than mean for PETF baseline, but this alone should not skew this analysis as most comparisons are based on paired measurements of the same individual, as, for example, the results shown in Figure 2. As baseline measurements were obtained after subjects donned the first garment, the higher baseline in CELF subjects conceivably resulted from ceramic particles influencing tcPO_2_ during the interval required to achieve stable measurements.

Another factor, the greater proportion of males to females in the CELF group versus PETF (62.5% vs. 45.2%) could have skewed the data analysis to show more of effects by CEL than is indeed the case, but as the men had lower baseline tcPO_2_ levels, and showed a moderately lower response to CEL than women, this seems unlikely.

If ST or core temperatures were higher under CEL than PET, then differences in tcPO_2_ might be due to heat induced vasodilation of the dermal microcirculation. Our study did not directly measure either core temperature or ST directly under the shirts, but the ST measurements from the subject's uncovered forearm make this explanation seem unlikely-ST generally fell throughout the protocol while tcPO_2_ generally rose. If temperature were the dominant factor influencing tcPO_2_, the later should have fallen in parallel with ST. Further, tcPO_2_ measurements entail heating skin to 44 °C, which should minimize the impact of variations in core or surface temperatures.

O_2_sat measurements differed when data from both sequence groups were combined. Ninety minutes after wearing CEL, mean O_2_sat was 98.5% for all subjects, significantly higher than the mean O_2_sat of 98.2% measured 90 minutes after donning PET. The pattern of change in O_2_sat with time in each group paralleled that observed with tcPO_2_. In PETF subjects, baseline O_2_sat rose slightly after 90 minutes of wearing PET, and then rose significantly more after 90 minutes wearing CEL With CELF subjects, baseline O_2_sat increased significantly over 90 minutes while wearing CEL, and after switching garments decreased significantly over 90 minutes while wearing PET. The modest similarity in the kinetic patterns observed with O_2_sat and tcPO_2_ measurements suggests they might be the result of the same ceramic particle influence. This raises the question of what is the underlying mechanism for the tcPO_2_ changes observed. Ceramic particles absorb heat (whether that be radiant, converted or conducted) emitted from the body, and then re-emit the thermal energy as IR (with a peak at 9.4 μm) back into the body. Re-emission occurs near the same wavelength as absorption, but may be at slightly a longer wavelength due to differences in temperature between the body and the fabric. This is not an energy neutral phenomenon, as the ceramics decrease the loss of infrared energy away from the body, those otherwise escapes through normal clothing.

The likely net result is increased absorption of FIR energy into the skin and underlying tissues. A recent study by scientists at Exponent Consulting compared the emissivity of PET fabric with or without CEL particles using sophisticated optical spectroscopic techniques. The intensity of infrared emission between 7.5 to 14 μm was 2.1% greater with fabric containing CEL particles (1.22% by weight) compared to fabric without ceramic particles [13]. This finding is consistent with the finding that the absorption co-efficient of the ceramic particles in the infrared spectrum was higher than the absorption co-efficient of pure PET fibers. In other words, the PET fibers are semi-transparent to infrared radiation, while the CEL particles are opaque. A follow-up study from the same group examined in more detail the influence of the ceramic particles on the infrared reflectance of the PET fabric, and measured the transmission, and absorption [14]. This analysis modeled the effects of the particles on infrared radiation incident upon the skin, as a function of wavelength, skin temperature, and ambient temperature, proportion of ceramic particles, air velocity, and influence of sunlight. The findings confirmed that the addition of ceramic particles to PET fabric leads to increased incidence of infrared radiation upon the skin at wavelengths longer than 4 μm, with a maximum effect at approximately 9.4 μm. The effect was attributed to increased absorption of infrared radiation at shorter wavelengths and reemission at longer wavelengths.

Increased emission of infrared from PET fibers with ceramic particles has the potential to interact with molecular and cellular structures by increasing the vibrational energy stored in chemical bonds, particularly in water clusters in cell membranes and cellular organelles. Perturbation of the vibrational energy of water clusters could affect the tertiary conformation of protein molecules tightly associated with this “nanostructured” water [15,16]. Low intensity far infrared (FIR-wavelength >14 um) lamps and topically applied (non-powered) FIR-emitting ceramic materials have been shown to induce cellular changes in vitro, and produce physiologic changes in both preclinical animal models and clinical studies. In none of these studies were the effects associated with significant changes in temperature, consistent with the low power associated with both far infrared lamps and non-powered ceramics in thermal equilibrium with skin (on the order of 0.1-1 mW/cm^2^ [3]. In vitro studies have found infrared radiation to inhibit cellular proliferation and be associated with increased reactive oxygen species; decreased production of intracellular nitric oxide and heat shock protein; inhibition of prostaglandin E2 synthesis; inhibition of kinase dependent nuclear signalling; and decreased production of inflammatory mediators and cell adhesion molecules [4,5,17-19].

Preclinical studies of infrared effects have shown inhibition of prostaglandin mediated inflammation in a rabbit arthritis model and delayed onset of muscle contraction induced fatigue [20]. Particular relevant to our findings are two reports in rats, one showing that infrared increased skin blood flow [21] and the other showed that infrared accelerated wound healing [7].

Three clinical studies have reported that blood flow was increased by infrared exposure from powered IR sources or non-powered ceramics [2,22,23] and one clinical study found alleviation of the symptoms of Raynaud's syndrome with ceramic impregnated gloves [24]. Other clinical studies with topically applied ceramic materials have demonstrated changes in body measurements (fat loss) [25,26], reduced dysmenorrhea [27], and improved lactation [28].

The increase in tcPO2 observed in this study likely is a consequence of increased oxygen availability in infrared illuminated tissue, possibly through a vasodilatory effect on the dermal circulation or, alternatively, effects on oxygen binding to hemoglobin. Although our understanding of the mechanism responsible for the effect of ceramic polyester composites on tcPO2 still incomplete, our data confirm that it is a real scientific phenomenon. Even without completely understanding the effect, it may be possible to design ceramic polyester composite garments or dressings that could improve wound healing, which is both sensitive to tissue perfusion and a critical problem for patients with diabetes. The recent decision by the US FDA that CEL garments will be regulated as medical devices and as general wellness products (http://www.medicaldevices-business-review.com/news/fda-determines-celliant-products-meet-criteria-as-medical-devices-260717-5882229) encourages clinical testing in multiple disease indications.

## Conclusion

The present study has added to the body of evidence that suggest that FIR-emitting garments can exert real measurable physiological effects, and deserve further study for medical indications. Especially the potential for ceramic-embedded fabrics to improve skin and wound perfusion has particular relevance to diabetes and warrants further study.

## Figures and Tables

**Table 1:** Subject demographics

Subject	% Male	Age (years)	Heights (inches)	Weights (ibs)	BMI
All (n=153)	54.3	38.3 ± 12.3	67.4 ± 3.9	167.2 ± 42.9	25.7 ± 5.5
CELF (n=180)	62.5^a^	37.9 ± 12.1	67.5 ± 3.8	171.3 ± 43.5	26.2 ± 5.7
PETF (n=73)	45.2^a^	38.7 ± 12.6	67.2 ± 4.0	162.8 ± 40.3	25.0 ± 5.2

a p=0.047, chi-square.

**Table 2:** Baseline physiologic parameters

Subject	tcPO_2_ (mmHg)	ST (°C)	%O_2_Sat
All (n=153)	64.9 ± 18.5	33.1 ± 1.0	98.0 ± 1.3
Male (n=83)	60.2 ± 18.5^a^	33.3 ± 0.9^d^	97.6 ± 1.7^f^
Female (n=70)	70.3 ± 17.2^a^	32.9 ± 1.2^d^	98.5 ± 1.1^f^
CELF (n=80)	66.4 ± 18.9	33.1 ± 0.9	98.0 ± 1.0
CELF Male (n=50)	62.9 ± 18.1^b^	33.3 ± 0.8^e^	97.6 ± 1.9^g^
CELF Female (n=30)	70.7 ± 18.1^b^	32.6 ± 1.0^e^	98.5 ± 0.9^g^
PETF (n=73)	63.9 ± 18.8	33.2 ± 1.1	98.1 ± 1.3
PETF Male (n=33)	56.2 ± 18.6^c^	33.3 ± 1.1	97.5 ± 1.3^h^
PETF Female (n=40)	70.0 ± 16.7^c^	33.3 ± 1.1	98.5 ± 1.3^h^

a p=0.0008

b p=0.059

c p=0.003

d p=0.01

e p=0.0009

f p<0.001

g p=0.023

h p=0.003

**Table 3:** Effects of Garments on ST and O_2_Sat

ST (°C)			
	Baseline	CEL 90 minutes	PET 90 minutes
All	33.1 ± 1.0	32.8 ± 1.0	32.6 ± 1.0
CELF	33.1 ± 1.0^a^	32.8 ± 1.0	32.4 ± 1.0a
PETF	33.2 ± 1.1^b^	32.9 ± 1.1b	32.8 ± 1.0
**%O_2_ Sat**			
	Baseline	**CEL 90 minutes**	**PET 90 minutes**
All	98.0 ± 1.3	98.5 ± 0.8^c^	98.2 ± 1.0^c^
CELF	97.9 ± 1.7^e^	98.5 ± 1.1 e^,^^f^	98.3 ± 1.0^f^
PETF	98.1 ± 1.3^d^	98.6 ± 0.8^d^	98.2 ± 1.2

a p<0.0001, paired t test

b p=0.064, paired t test

c p<0.0001 paired t test

d p=0.0001, paired t test

e p=0.0002, paired t test

f p=0.003, paired t test
